# Vaso-Occlusive Pain Management in a Patient With Sickle Cell Disease Associated With Moyamoya Syndrome

**DOI:** 10.7759/cureus.23667

**Published:** 2022-03-30

**Authors:** Drezelle Mills, Annie Wang, Iya Dubson

**Affiliations:** 1 Internal Medicine, St. George’s University School of Medicine, St. Georges, GRD; 2 Internal Medicine, Kings County Hospital Center, Brooklyn, USA; 3 Internal Medicine, State University of New York Downstate Health Sciences University, Brooklyn, USA

**Keywords:** pain management, vaso-occlusive pain, hypotension, sickle cell disease, moyamoya syndrome

## Abstract

Moyamoya syndrome in a sickle cell disease patient may be a difficult task to manage in the setting of a vaso-occlusive pain crisis. Maintaining stable blood pressure is necessary to prevent stroke as both hypertension and hypotension can be detrimental to the patient, leading to hemorrhagic and ischemic stroke, respectively. Opioid management for pain control in such patients must be taken into consideration. Because every patient is unique, opioid regimens should be optimized to relieve patients’ specific pain while also practicing non-maleficence in preventing hypotension and strokes.

## Introduction

Moyamoya is a Japanese term translated as “puff of smoke” which is used to describe the pattern of neovascularization of tiny blood vessels in the brain compensating for blocked internal carotid arteries. Moyamoya disease (MMD) is defined by this radiographic finding, while moyamoya syndrome (MMS) is the presence of MMD in addition to other comorbidities such as sickle cell disease, Down’s syndrome, or neurofibromatosis [1]. MMD typically affects children in the first decade of life (average age of nine years) and rarely adults (around 30-40 years of age) [2].

Throughout North America, little data are available on MMS associated with sickle cell disease. MMS is exceedingly rare with an estimated incidence of 0.086 per 100,000 persons in the western demographic of the United States, specifically in Washington and California [3]. Over a 10-year period, compared to Caucasians, the disease incidence was higher among Asian Americans with an incidence rate ratio of 4.6, followed by African Americans with an incidence rate ratio of 2.2 and Hispanics with an incidence rate ratio of 0.5 [3]. This case report aims to discuss an unusual case of MMS in a 50-year-old African American female with sickle cell disease and describe a method of treatment for vaso-occlusive crisis with chronic pain while considering unfavorable vital signs.

## Case presentation

A 50-year-old female with a medical history of sickle cell disease, seropositive rheumatoid arthritis, and MMS complicated by multiple strokes with residual right-sided weakness, cognitive impairment, and seizures presented to our emergency department with severe pain in her chest, lower back, and bilateral legs, more intense than her typical acute vaso-occlusive pain episodes. The patient’s mother reported that home doses of combination oxycodone and acetaminophen did not relieve her pain. Initial vitals were notable for a temperature of 36.4°C, blood pressure of 92/44 mmHg, pulse of 98 beats per minute, and oxygen saturation in the 70s. Labs were significant for hemoglobin of 3.3 g/dL (baseline 5 g/dL), lactate dehydrogenase 667 U/L, and reticulocyte 15%. On further evaluation and workup, the patient was also found to have a urinary tract infection (UTI). She was then admitted to our general medicine floor for management of her vaso-occlusive crisis secondary to UTI.

According to the patient’s mother, her primary caregiver, she was diagnosed with sickle cell disease at age three. She had her first stroke at eight years of age, was diagnosed with MMS at ten years, and subsequently had multiple strokes. The patient’s home pain regimen included oxycodone/acetaminophen 5-325 mg taken as needed for pain. Other home medications included aspirin, hydroxychloroquine, levetiracetam, and topiramate.

During the hospital course, the patient was initially started on hydromorphone 0.5 mg intravenously every four hours as needed for pain. Given the patient was opiate-naïve, she developed multiple episodes of hypotension and bradycardia with systolic blood pressure (SBP) of 54-105 mmHg, diastolic blood pressure (DBP) of 34-74 mmHg, and heart rate of 48-98 beats per minute. To avoid further hypotension, bradycardia, and respiratory depression, hydromorphone was discontinued, and the pain regimen was adjusted to oxycodone-acetaminophen 5-325 mg every six hours. Vitals returned to the baseline range after adjustment of medication, SBP ranged between 107 and 118 mmHg, DBP ranged between 61 and 71 mmHg, heart rate ranged between 61 and 71 bpm, and the pain was controlled. Other considerations included avoiding lidocaine transdermal patches because even minimal lidocaine absorption into the circulation could be hemodynamically detrimental in the patient. Baclofen was also added to address musculoskeletal pain.

**Figure 1:**
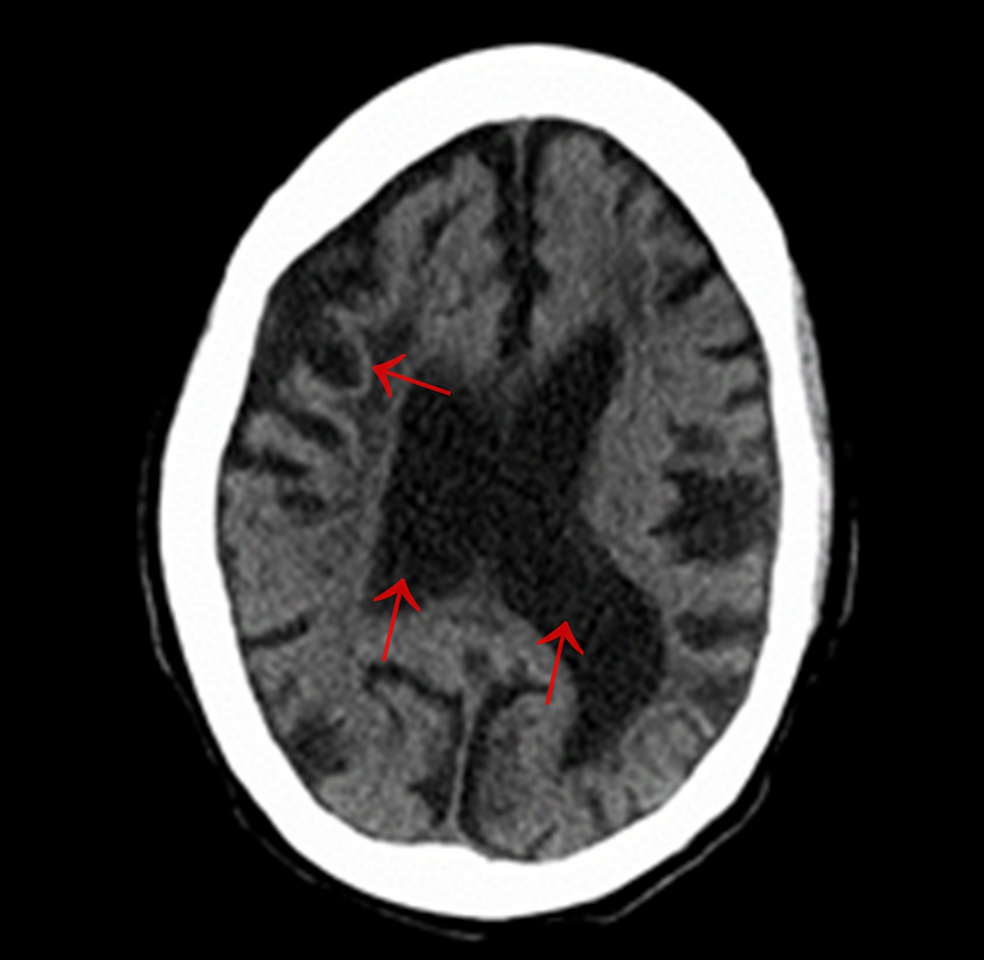
Multifocal large regions of encephalomalacia (after several events of cerebral infarction) with prominence of the lateral ventricular system.

**Figure 2:**
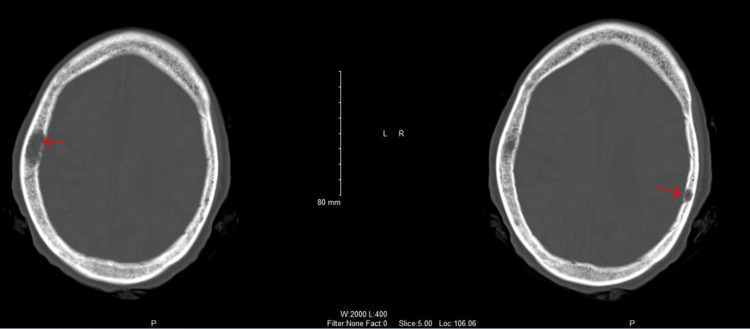
Bilateral parietal bone lytic lesions containing soft tissue.

In addition to pain management, the patient was given gentle intravenous fluid hydration and a total of three units of packed red blood cells with improvement of blood pressure and hemoglobin levels to 6.6 g/dL at baseline on discharge. Her UTI was also treated with antibiotics. The patient remained hemodynamically stable on the adjusted pain regimen without signs of acute stroke throughout her hospital course. Computed tomography scans of the head without contrast are shown in Figures 1, 2.

## Discussion

In patients with sickle cell disease, MMS can be seen in about 20-30% of cases and can be detected using brain angiograms [4]. Moyamoya predisposes patients to both ischemic and intracranial hemorrhagic strokes [4]. The long-term management of MMD and MMS primarily involves stroke prevention. During an acute stroke, standard therapy involves the use of antithrombotic agents and measures taken to reduce intracranial pressure to improve blood flow to the brain. However, as moyamoya patients are at risk for developing both ischemic and hemorrhagic strokes, the use of anticoagulation is often not preferred. The mainstay of treatment includes pharmacotherapy with antithrombotics and revascularization surgery [5].

Regarding antiplatelet therapy, studies on the benefit of antiplatelet agents in MMD are limited; however, antiplatelet therapy with aspirin or cilostazol is commonly incorporated into practice. In a study using the Korean National Health Insurance database, antiplatelet therapy was found to reduce mortality in patients with MMD compared to those who did not use antiplatelet therapy. In particular, cilostazol provided more survival benefit compared to aspirin, clopidogrel, and other antiplatelet agents [6].

To prevent subsequent strokes, surgical revascularization can also be considered in some cases. In a study monitoring the features and outcomes of MMD in North America, after the initial symptoms of MMD, mostly ischemic stroke, the rate of recurrent strokes was decreased by 17% in those who underwent moyamoya revascularization compared to 65% in those who did not [7].

The complications of moyamoya angiopathy, such as seizures and chronic headaches, should also be clinically managed with antiepileptics and analgesics [5]. Concerning analgesics, oxycodone like many other opioids, leads to hyperpolarization and decreased neuron signaling, causing central nervous system depression. Though uncommon, these changes can lead to a drastic drop in blood pressure [8]. In our case study in an opioid-naïve patient, oxycodone was effective in alleviating vaso-occlusive pain while maintaining adequate blood pressure compared to hydromorphone given initially.

Other analgesics can be considered such as tapentadol and morphine. In a study involving 1,464 patients over 15 weeks that evaluated blood pressure and heart rate changes between oxycodone and tapentadol, oxycodone was found to have a more significant effect in decreasing systolic and diastolic blood pressure from patients’ baseline. The study concluded that the management of pain is safer using tapentadol in patients with chronic pain and hypertension [9]. In addition, morphine, one of the most common opioids, can be used as an alternative. However, morphine has increased histamine release, leading to greater side effects of pruritus and gastrointestinal dysfunction [10].

## Conclusions

MMD is an extremely rare disease, especially in North America and non-Asian populations. In the management of vaso-occlusive pain with opioids in patients with sickle cell disease and MMS, careful consideration should be taken to monitor blood pressure. Hypotension should be avoided to prevent decreased brain perfusion, especially in patients with MMS who are at increased risk of stroke. In the event of continuous hypotension due to oxycodone, patients can be switched to tapentadol if the pain is responsive. Other long-term and definitive management can be performed such as revascularization surgery to reduce subsequent strokes.
